# Parallel Solutions for Voxel-Based Simulations of Reaction-Diffusion Systems

**DOI:** 10.1155/2014/980501

**Published:** 2014-06-12

**Authors:** Daniele D'Agostino, Giulia Pasquale, Andrea Clematis, Carlo Maj, Ettore Mosca, Luciano Milanesi, Ivan Merelli

**Affiliations:** ^1^Institute of Applied Mathematics and Information Technologies, National Research Council of Italy, Via de Marini, 16149 Genoa, Italy; ^2^Genetic Unit, IRCCS Saint John of God, Clinical Research Centre, Via Pilastroni 4, 25125 Brescia, Italy; ^3^Institute of Biomedical Technologies, National Research Council of Italy, Via Fratelli Cervi 93, 20090 Segrate, Milan, Italy

## Abstract

There is an increasing awareness of the pivotal role of noise in biochemical processes and of the effect of molecular crowding on the dynamics of biochemical systems. This necessity has given rise to a strong need for suitable and sophisticated algorithms for the simulation of biological phenomena taking into account both spatial effects and noise. However, the high computational effort characterizing simulation approaches, coupled with the necessity to simulate the models several times to achieve statistically relevant information on the model behaviours, makes such kind of algorithms very time-consuming for studying real systems. So far, different parallelization approaches have been deployed to reduce the computational time required to simulate the temporal dynamics of biochemical systems using stochastic algorithms. In this work we discuss these aspects for the spatial TAU-leaping in crowded compartments (STAUCC) simulator, a voxel-based method for the stochastic simulation of reaction-diffusion processes which relies on the S*τ*-DPP algorithm. In particular we present how the characteristics of the algorithm can be exploited for an effective parallelization on the present heterogeneous HPC architectures.

## 1. Introduction

Systems biology provides a general framework for integrating pharmacology and genetics through mathematical models [1]. In this context, there is an increasing recognition that stochastic processes regulate highly predictable patterns of gene expression in developing organisms, but the implications of stochastic gene expression for understanding phenomena such as genomics mutations and copy number variations remain largely unexplored [2]. Therefore computational tools that consider biological noise are suitable for pharmacogenomics research, towards personalized medicine [3].

In particular, gene expression is an inherently stochastic process: genes are activated and inactivated by random association and dissociation events, transcription is typically rare, and many proteins are present in low numbers per cell. If large numbers of identical events occurred in the same cell and they were statistically independent, relative fluctuations could be ignored and deterministic rate equations would suffice to describe dynamics. But in several processes numbers are not large and events are not independent. Active genes are often present in a single copy, mRNAs can be rare, and most proteins are present in less than 100 molecules per bacterial cell. Substrates, enzymes, and regulatory molecules can also fluctuate and further randomize expression rates [4].

Moreover, the integration of synthetic and cell-free biology has made tremendous strides towards creating artificial cellular nanosystems using concepts from solution-based chemistry, where only the concentrations of reacting species modulate gene expression rates. However, it is known that macromolecular crowding, a key feature in natural cells, can dramatically influence biochemical kinetics via volume exclusion effects, which reduce diffusion rates and enhance binding rates of macromolecules. The macromolecular crowding can increase the robustness of gene expression by integrating synthetic cellular components of biological circuits and artificial cellular nanosystems. Furthermore, a negative feedback loop and the size of the crowding molecules can fine-tune gene circuit response to molecular crowding [5].

The simplified representation of intracellular reactions with homogeneous approaches may be inadequate when the observed properties of the real system are also the consequence of macromolecular crowding. Molecular crowding is a natural state of cells in which their intracellular environments are densely packed with macromolecule [6, 7]. This crowding is absent in the solution-based chemistry approaches that are typically used in synthetic genetic systems. Molecular crowding can cause volume exclusion effects that reduce diffusion rates and enhance the binding rates of macromolecules [8], leading to a fundamental impact on cellular properties such as the optimum number of transcription factors [9], the dynamical order of metabolic pathways [10], and nuclear architecture [11]. In such situations, a more accurate representation is achievable by using modelling approaches based on reaction-diffusion (RD) systems taking into account the geometry of the space and the effect of crowding elements.

Noise of biological systems and macromolecular crowding can be faced using stochastic reaction-diffusion models with crowding objects that influence reactive and diffusive events. A system of this type can be simulated using the S*τ*-DPP algorithm [12]. Due to the necessity of simulating the system a large number of times to obtain a satisfactory statistical description of its dynamics and considering that each single simulation of the algorithm is still time-consuming; in this work we present two parallelizations of the spatial TAU-leaping in crowded compartments (STAUCC) simulator (a C implementation of the S*τ*-DPP algorithm [12]), one for distributed memory systems, such as clusters for high performance computing (HPC), and the other for devices based on the CUDA architecture (http://www.nvidia.com/object/cuda_home_new.html).

This work represents an extension of [13, 14], where we presented a brief description and the preliminary results of, respectively, the parallelization based on the message passing interface (MPI) standard (http://www.mpi-forum.org/) and the CUDA-based implementation of STAUCC. In particular, here we describe in detail the two implementations, which have been improved in the view of the matured experience, with an in-depth comparison of their performance on a real-world test case.

The paper is organized as follows. In Section 2 we present related works. In Section 3 we resume the cardinal points of the S*τ*-DPP algorithm used in STAUCC, whose MPI and CUDA implementations are described, respectively, in Sections 4 and 5. In Section 6 the gene regulatory network to which we applied the STAUCC simulator is presented; in Section 7 the experimental results are discussed, followed by the conclusions and future work drowned in Section 8.

## 2. Related Works

Two broad classes of methods for stochastic RD exist: particle-based and voxel-based methods. Particle-based methods compute the Brownian motion of individual particles (e.g., MCell [15], Smoldyn [16], and GridCel [17]), while voxel-based methods calculate changes in the number of molecules occurring in small well-stirred compartments in which the space modelled is partitioned (e.g., STEPS [18], MesoRD [19], and NeuroRD [20]).

Particle-based modelling is very computationally intensive, because it means to solve the equation of motion for each molecule of the system, which is appropriate only to compute the behaviour of few molecules at best [21]. On the other hand, voxel-based approaches do not model the motion of each single protein but have enough accuracy to capture how spatial gradients influence system dynamics.

So far, different parallelization approaches have been deployed to reduce the computational time required for the simulation of biochemical systems modelled using a voxel-based approach and simulated employing stochastic algorithms. Most of the proposed solutions are based on the use of an embarrassingly parallel approach to run several instances of simulation of the model at a time, maintaining the simulation algorithm sequential. A typical example is given by the exploitation of distributed platforms, such as with Grid computing [22] and multicore CPUs [23].

An efficient parallelization of the algorithm itself, which improves the execution time of each specific simulation, can exploit more effectively the available computational resources, allowing to reach a higher computational efficiency. An MPI implementation of STAUCC which parallelizes the S*τ*-DPP algorithm [12] was presented in [13].

As regards the use of accelerators, in the literature some works considered the use of graphics processing units (GPUs) [24] and also field-programmable gate arrays (FPGAs) [25]. We presented a GPU based implementation of STAUCC in [14] that in particular differs from [24] because (1) we consider the volume of the molecules, which results in more synchronization operations for checking, at each time step, the free space left in the membranes, and (2) because the Fermi architecture we exploited presents different capabilities with respect to those exploited for the optimization strategies they proposed.

## 3. The Spatial TAU-Leaping in Crowded Compartments (STAUCC) Simulator

The spatial TAU-leaping in crowded compartments (STAUCC) simulator implements the S*τ*-DPP algorithm [12], whose pseudocode is presented in Algorithm 1. STAUCC exploits a *τ*-leaping method to stochastically simulate the evolution of biochemical systems, which can be defined as a set of chemical reactions (i.e., rules), and supports the modelling of spatial effects due to molecular crowding.

A system is defined as a set of compartments containing a multiset of objects or molecular species. Compartments can contain other compartments, in order to describe nested configurations of the biological system (e.g., nucleus inside cytoplasm). At a given time instant *t*, each object is associated with only one compartment. The state of the system at time *t* is given by the number of molecules of each species inside each compartment.

For each compartment there is a set of rules describing both the biochemical reactions and the molecular diffusion gradients. A reaction rule substitutes the molecules specified in its left-hand side (reactants) with the molecules specified in its right-hand side (products). A diffusion rule moves reactants from the current compartment to the compartment specified in the right-hand side.

In case a reactant that is involved in more than one rule is present in little amount, the occurrence of a rule before the other(s) may prevent the other(s) rule(s) from happening and vice versa. In such cases, the system can evolve following different dynamics according to the order in which rules occur. The reactions that involve potential limiting reactant(s) are defined as “critical reactions”: these rules must be executed sequentially in order to avoid negative species population.

Each rule is associated with a propensity function (stochastic reaction rate), defined as *a* = *c* · *h*, where *c* is the stochastic constant associated with the rule and *h* is the number of possible combinations of reactants appearing in the left-hand side of the rule [26]. However, the novelty of the S*τ*-DPP algorithm is that it takes into account also the size of compartments and molecules involved in the system, in order to describe the effect of crowding on the rate of cellular processes (for more details see [12]). This is the reason why propensity functions of reactions are computed by also considering the amount of free space in the current compartment. The free space of a compartment *i* at time *t* is defined as *V*_*f*,*i*_(*t*) = *V*_*i*_ − *V*_*i*,*o*_(*t*), where *V*_*i*_ is the compartment size and *V*_*i*,*o*_(*t*) is the space occupied by molecules and compartments existing inside compartment *i* at time *t*. Propensity functions of reactions of order greater than one (e.g., A + B → R) are functions of the free space: *a* = *c* · *h* · *V*_*f*,*i*_^(−1)^. Thus, the lower the free space, the higher the probability of reactive collision. Conversely, propensity functions of reactions of order less than two (e.g., A → R) and diffusive events are not function of the free space of the current compartment. However, diffusive events toward compartments with not enough free space are prohibited.

At each iteration, a time increment *τ* is computed independently inside each compartment (line 3 of the pseudocode), on the basis of its current state. Then, the smallest time increment is selected among those computed (line 4: in a parallel implementation, this must be done after a first synchronization of the computation in all compartments) and shared by all compartments as the global time increment on which to synchronize their evolutions. This time increment indeed is used to evaluate the evolution of the entire system, as specified by the standard *τ*-leaping algorithm [27], which requires the generation of a Poisson random variable to characterize the stochastic behaviour of the model.

First of all, this time increment determines the way to proceed in the current iteration: SSA-like evolution, *τ*-leaping evolution with noncritical reactions only, or *τ*-leaping evolution with noncritical reactions and one critical reaction. Then, the same time increment is used, together with the computed propensity functions, to sample the number of reactions to be executed in each compartment, according to the chosen modality (line 5).

A second synchronization point among the compartments is now necessary for a consistent application of the extracted rules, which implies to consider a new time increment *τ* (line 6) or the exchange of molecules among the compartments (line 7(b)).

Finally, before stepping over the next iteration of the simulation, to ensure that the system has not ended up in an unfeasible state (i.e., in at least one compartment there are more molecules than the maximum number containable in it), the free space left inside each membrane is checked, and until it results negative in at least one membrane (third synchronization point), the time step is repeatedly reduced by half and another set of rules is extracted and applied to the system (line 9).

## 4. The MPI Parallel Implementations of the STAUCC Simulator

STAUCC presents three possible levels of parallelization. The simplest one is related to the stochastic nature of the simulation; that is, many independent instances of STAUCC, from several dozens up to several thousands, can be executed in an embarrassingly parallel way, using different cores/nodes of a local HPC system, or distributed infrastructure, such as the Grid, in order to achieve a statistically accurate result.

The second one is related to the execution of each single simulation, which can exploit several parallel processes by assigning each of them to one or more membranes. In this case the steps 2, 3, 5, 7(a), and 8 of the algorithm are executed in parallel, with steps 4, 6, 7(b), and 9 representing communications-synchronization points, to be performed in a collaborative way among the parallel processes.

The third parallelization paradigm is related to the evaluation in parallel of the propensity function, represented by step 3(a) which, for example, can be performed by one thread for each rule.

Considering that many biological systems can be modelled using a relatively low number of reactions (e.g., in the order of tens for central metabolism and cell cycle regulation) and considering that in each time increment *τ* just few of them are fired together, it is a reasonable choice to focus the parallelization on the first two levels described above. This choice is also supported by the consideration that the number of compartments in which the space is discretized is usually much greater than the number of rules of the biochemical system; that is to say, a space-based paradigm of parallelization can provide more flexibility and scalability than a rule-based approach. Therefore, we focused on the first two levels of parallelization and we implemented it using MPI; that with respect to OpenMP allows exploiting of multiple nodes of a cluster.

## 5. The CUDA Implementation of STAUCC

Also the CUDA implementation of STAUCC exploits the same two-level parallelism, which means that it is possible to launch concurrently several simulations of the model on a GPU using different blocks, while, in each simulation, the temporal evolution of the state of all compartments is also computed in parallel by threads in a block. Indeed, as already said, in the STAUCC algorithm the space is partitioned in smaller regions or compartments, and most of the steps of the algorithm can be performed by parallel threads.

We developed a single CUDA kernel in which the thread blocks carry out independent simulations of the model, given an initial condition of the system and a seed for the generation of the chain of random numbers used by the algorithm throughout the computation of the system's dynamics. Inside each block, the workload is balanced among the threads assigning to each of them the computation of the evolution of the most possible equal number of compartments. In this way, for any given number of compartments in the system, we are free to choose the number of threads per block which best fits the GPU architecture and then launch number of blocks according to the number of simulations we want to perform, the number of stream multiprocessors (SMs) provided by the GPU device, and the amount of its memory.

Inside the thread block, at each iteration of the algorithm (or time frame), three communication/synchronization points are required (see the previous section) as in the MPI-based parallelization. The synchronization operation on the present CUDA architectures is possible only among the threads of a block, therefore the structure of the algorithm must take this aspect into account.

A fundamental problem in developing parallel applications is the memory management. In a first version, we placed in the device shared memory those variables which must be visible to all threads, either because involved in reduce operations (e.g., the computed time increment *τ* and the free space for each compartment) or because they are changed by the threads of other compartments (e.g., the number of molecules), and in the device constant memory the fixed parameters of the system (e.g., the stochastic constants, the sizes of molecules and compartments, the left-hand and right-hand side of rules, etc.).

However, we then decided to place all the variables in the device global memory because of at least two reasons. The first one is the principal reason and it is because our main goal is to provide a simulator which can deal with a large number of membranes, as for real-world simulations. Keeping in the shared or constant memory even a single location (integer or floating point, 4 bytes anyway) per membrane (i.e., an array of sizes equal to the number of membranes) imposes too strict limits to the size of the systems which is possible to simulate. For example, if the shared memory is 48 Kbytes (the maximum amount for each stream multiprocessor of an Nvidia GPUs available nowadays on the market) and we want to run concurrently 16 simulation instances (i.e., we launch a grid of 16 blocks), then the maximum number of membranes each block can process would be 48 Kbytes/(16 × 4) = 768 bytes, rather small for our purposes. The second reason is related to the improvements in the CUDA architecture and programming model. In [24] the smart use of shared and constant memories was of fundamental importance because the main memory of a graphic card based on the Tesla architecture is not cached, while this feature is present on the more recent Fermi and Kepler architectures. Moreover the smart and transparent memory management is a key topic for the development of the CUDA programming model, as demonstrated by the Unified Memory (http://devblogs.nvidia.com/parallelforall/unified-memory-in-cuda-6/) feature introduced CUDA 6.0. Therefore sophisticated strategies for the use of registers and the various kinds of memories have to be properly evaluated on the basis of the actual application behaviour, because they may have little importance in a short time.

In a second version of the implementation, to parallelize the memory allocation/deallocation phases also, we allocated/released dynamically the arrays in the device heap memory, doing a per-block allocation/deallocation for the arrays that before were put in the shared memory (i.e., the arrays common to all threads in a block) and a per-thread allocation/deallocation for the others. However, in this way the threads access very sparse memory locations, thus causing a very bad use of the L1 cache, and this fact globally worsens the execution time, despite the speedup of the allocation/deallocation phases.

The best solution achieved for the memory management of this application therefore is to allocate through a unique host-side CUDA API call such as* cudaMalloc* all the one-dimensional arrays used by all threads in all blocks, and inside the computational kernel we declared pointers to the memory regions reserved to the thread blocks to reduce address arithmetics. The arrangement of the variables in memory at this point must be such that the threads access memory locations with as much unitary stride as possible. For example, if we need to store for each membrane the amount of molecules for each species inside it and we have to deal with M membranes, S species per membrane, and T threads per block (supposing for simplicity that M is a multiple of T), a good solution is to partition the global one-dimensional array allocated (which will be of size M × S) into S subarrays of M elements each and to further divide the subarrays into groups of T adjacent elements. In this way we can access the variables in a fully coalesced way with an external loop over the S species and an internal loop over the membranes associated with each thread (M/T): at each iteration, the T threads in the block will access a contiguous group of T cells in memory. We minimize the calls to device functions inside the kernel limiting them only to those quite long parts of code repeatedly executed, to avoid further overheads.

Regarding the stochasticity of the algorithm, we used the device APIs of the CURAND library (https://developer.nvidia.com/curand) provided within the CUDA Toolkit (production release 5.5). In particular, we used the pseudorandom number generator based on the XORWOW algorithm. As suggested in the library guide, for the highest quality parallel pseudorandom number generation, each experiment (in our case, each block) has been assigned a unique seed, and within an experiment each membrane has been assigned a unique sequence number (i.e., a random state). Thus during the simulation each thread extracts the chain of random numbers from the random state of the membrane of which it is computing the evolution.

Having enclosed in a kernel the computation of the dynamic evolution of the system, we avoid any communication between host and device during the simulation. Yet it is true that, having quite all the variables of the system stored in the global memory, it could be possible to access them if necessary from the host with little effort. In fact we should simply return the kernel, keeping track of the current time frame, and then launch another kernel starting from the step where we stopped, but this would considerably slow down the computation time. Thus, the state of the system (the time frame plus the number of molecules in each compartment) is copied every *N* iterations in a preallocated buffer in the device global memory that is read back from the host after the kernel returns.

## 6. Simulating a Gene Regulatory Network with STAUCC

It is well known in biology that gene expression can be regulated by a different number of factors, which inhibit or promote gene expression under different conditions [28, 29]. Regulatory factors occur in low copy number and, in fact, noise plays a relevant role in gene expression [30]. A pivotal element in the interactions between regulatory factors and DNA is given by the diffusion of regulatory factors within the cell nucleus [31], which is an environment crowded by chromatin. Taken together, these considerations underline the need for a stochastic RD approach to model gene expression, possibly considering also the crowding effects created by the presence of the DNA in the nucleus.

**Figure 1:**
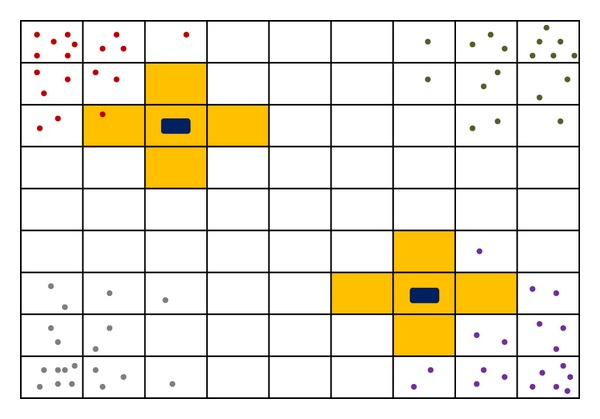
Schematic representation of the space domain. The 2D grid of compartments represents a section of the nucleus in which two genes, G_1_ and G_2_ (black rectangles), are located. Four regulatory factors (represented with different colour circles) can diffuse within this environment. Compartments filled in yellow have a lower free space, which model the macromolecular crowding due to chromatin. The representation is not in scale with actual sizes used in simulations.

**Figure 2:**
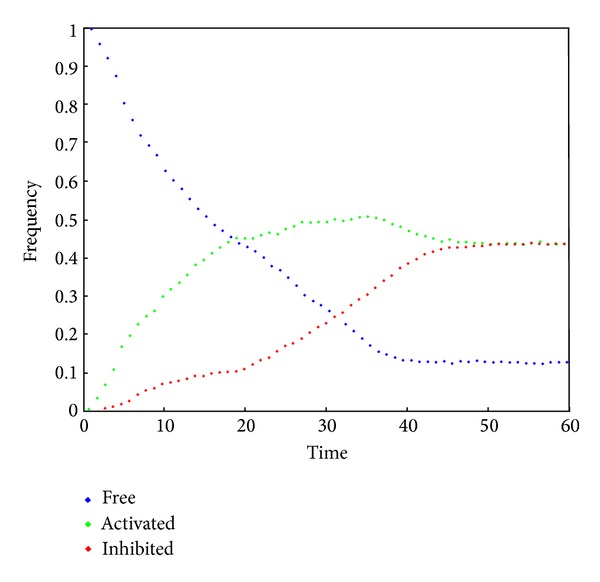
Evolution of G_1_ state probability starting from an initial condition in which an activator is placed closer to the gene with respect to an inhibitor. The vertical axis represents the G_1_ frequencies that have been found for each state in relation to the simulation time (horizontal axis).

In order to provide a realistic use case for the STAUCC we have defined a model of a gene regulatory system with explicit consideration of space and crowding. The nucleus is represented as a two-dimensional grid composed of a finite number of squared compartments of the same size, as shown in Figure 1. Six types of objects are considered: four regulatory factors, F_1_, F_2_, F_3_, and F_4_, and two genes, G_1_ and G_2_. While genes are modelled as static objects, regulatory factors diffuse freely. The compartments in which the genes are placed, as well as the adjacent compartments, have lower free space, in order to represent the crowding due to the presence of DNA and DNA-interacting proteins. Factors that reach these compartments can bind genes in order to activate or inhibit their expression (see, e.g., the evolution shown in Figure 2). Therefore each of the two genes can be in one of the following three states: free, a state in which a factor can bind the gene; active, in case an activation factor binds the gene; inhibited, in case an inhibition factor binds the gene. A total of sixteen reaction rules have been defined in order to describe the possible interactions between genes and regulatory factors, as listed in Table 1. In our model, the stochastic constants represent (i) gene regulatory factor association/dissociation constants and (ii) regulatory factors diffusion coefficients. Diffusion coefficients have been set higher (i.e., faster) than association/dissociation constants of two orders of magnitude. The fact that the time scale of diffusion processes is much lower than the time scale of reaction processes is crucial for maintaining valid the well-stirred assumption within each compartment [12]. The model has been simulated considering an increasing number of compartments, that is, 256, 1024, and 4096.

At the beginning of a simulation all the objects representing a regulatory factor F_*i*_ are placed at corners of the lattice, while the two genes are placed in two inner compartments (see again Figure 1). An example of system dynamics is shown in Figure 2.

## 7. Performance Evaluation

Two are the resources adopted for the simulation. The first one is a cluster composed of 18 nodes equipped with two 4-core Intel Xeon E5345 CPUs, 16 GB of Ram, linked together via an Infiniband QDR network.

The second one is a subset of three heterogeneous nodes of a cluster equipped with two 6-core Intel Xeon E5645 CPUs, 32 GB of RAM, and one different CUDA device: a GTX 580, a K20, and a GTX-Titan. We exploited in both cases also the Intel Cluster Studio XE 2013 suite, in particular the C compiler and the MPI library implementation.

The execution times of all the three STAUCC implementations (sequential, MPI, and CUDA) are proportional to the number of iterations, that is, the number of times the steps 3–9 are performed until the termination criteria are satisfied. This depends on both the initial conditions (i.e., the initial state and parameter values) and the chain of generated stochastic numbers. In general, a higher number of iterations are required whenever the system dynamics are fast, that is, when many reactions occur and the system state (number of molecules) is significantly modified: in this scenario in fact small *τ* values will be required to satisfy the leap condition and any iteration will represent a short interval of time.

It is worth noting that, due to the use of randomly generated numbers, two runs with the same initial conditions are likely to have a different evolution and thus a different number of iterations. For example, the execution time of the sequential implementation of STAUCC for the 256-compartment system can vary between 43.73 seconds for 6,244 iterations (wall clock time) and 2,214.23 seconds for 427,156 iterations. This large variation is the consequence of the specific sequence of states the system will assume during the simulation on the basis of the assigned random value. In fact, states associated with large propensity function values and with reactions that determine significant variations of propensity function values will require shorter time increments and, therefore, a larger number of iterations.

The time for data acquisition depends on the size of the system description that is lower than 1 MB and therefore negligible, while the output time depends on a user-defined parameter that specifies the sampling frequency of the system status. By sampling every 100 iterations, in the slowest case, the result corresponds to more than 100 MB of output data, while the production of only the final state of the system results always in a size of a few MB.

These differences in output size and therefore in the time that is required for building the output can be a problem in the analysis of the scalability of the system. Therefore in the following we analyse the performance of the three implementations by comparing the average time for executing a single iteration (hereafter called* TimePerStep*).

### 7.1. The MPI Implementation of STAUCC

Each node of the first cluster has 8 cores, and therefore we considered six execution patterns: 2, 4, and 8 parallel processes running on a single node, to test the scalability within a single node, and 16, 24, and 32 parallel processes spread among 2, 3, and 4 nodes, to test the impact of the communication overheads. Results are shown in Table 2.

We can see how the variation of the number of compartments used to model the nucleus space affects the scalability of the algorithm. With 256 regions the use of all the 8 cores of a node is the most effective parallelism degree: in fact if we execute two instances of STAUCC on two nodes, we achieve, globally, a speedup of 3.5 × 2 with respect to 3.2, that is, executing a single simulation with 16 cores. The usage of two nodes is suitable only with at least 4096 compartments, while the maximum parallelism degree is achieved for the largest simulation, that results in a speedup of about 20 using 4 nodes, very close to the speedup of about 24 (i.e., 6.10 × 4, the speedup achieved by using all the 8 cores of a node) achievable by executing a single instance per node.

These performance values are due to the fact that increasing the number of processes and exploiting more than one node has the consequence that the communication times overcome the computation times, and this is also with a fast interconnection as Infiniband. As said before, each iteration requires 3 communication operations among all the parallel processes, and this limits the achievable performance. In particular the smallest time increment and the correctness check were implemented using the* MPI_Allgather* function, while the transfer of molecular species is performed by an* MPI_Reduction* followed by an* MPI_Scatterv* function call. This has the advantage of reducing the data transfer of the data with respect to the use of a single* MPI_Allgather*: in the largest case in fact the system state has a size of 1 MB.

By analysing the trace data of the cases 8 and 16 for 4096, we can see that using two nodes the time for the communication operations is about 4 times more than the time needed to perform the computation. On the contrary if all the processes are on one node, the ratio is about 2 times. It is worth noting that this last result is achievable only if an optimized implementation of the MPI library is used, as with the Intel one. General purpose implementations as MPICH2 1.2.5 in fact provide lower performance and it is better to implement the same parallelization strategy with the OpenMP paradigm.

Two considerations hold true. At first it is worth noting that the average execution time for a single 4096-membrane simulation is of about 6 hours in the sequential version, while with our parallel implementation we are able to get the results in 18 minutes using four nodes. This means that the parallel implementation using clusters of ×86 multicore processors is effective but the parallelism degree has to be properly tuned considering the size of the system to simulate. The second one is that, due to the stochastic nature the analysis STAUCC provides, the goal can be to obtain the results of many different simulations in the shortest possible times. In this case the most effective solution is to exploit a hybrid MPI-OpenMP model where the MPI processes are responsible for orchestrating different stochastic simulations, that is, for the subdivision of the generated random numbers among them, while each simulation is performed within a single node using all the cores with parallel threads provided by the OpenMP paradigm.

### 7.2. The CUDA Implementation of STAUCC

The STAUCC parallel simulator presented in this paper has been tested on three different Nvidia devices. The GeForce GTX 580 device, based on the CUDA Fermi architecture, was selected because it is equivalent in terms of the price for their acquisition to the Intel Xeon E5645 CPU equipping the nodes of the second cluster, while the K20 and GTX-Titan, based on the CUDA Kepler architecture, are, after the recent K40, the most powerful CUDA-based devices produced by Nvidia in 2014.

We simulated the system by distributing the workload among a block of 32 threads for the GTX-580 device (i.e., one warp per block), with 128 for the other two devices, because this resulted in the more effective configuration. With these problem sizes in fact it is difficult to exploit all the 192 cores and each thread computes in any case the evolution of many compartments.

For each system size, we launched concurrently on the GPU an increasing number of stochastic simulations; that is, we launched the simulation kernel with an increasing number of blocks (as explained before, the evolution of a simulation instance is computed entirely by a single block of threads independently from the others). As larger systems occupy more space in the global memory of the device and we avoid any communication between host and device during the simulation (which is performed running a single kernel as explained), the maximum number of simulation instances that is possible to launch decreases as the number of compartments increases. For this reason, the kernel can compute a maximum of 256 simulation instances for the smallest system on the small GTX-580 device and between 64 and 84 for the largest one when considering the three different devices.

As a consequence, the speedup of the STAUCC simulator is computed considering the average time for an iteration of the model simulation (*TimePerStep*) and dividing this quantity in the sequential simulation by the same quantity in the parallel simulation: in this case, the total number of iterations performed is the sum, over the total number of simulations launched, of the number of iterations computed in each instance of simulation.

Results are shown in Table 3 for the simulation of the three systems on the GTX-580 and in Table 4 for a comparison of the results on the 4096 system among the three devices. The first consideration is about the memory allocation/deallocation times. They maintain quite constant values when varying the system size in the considered range both in the sequential and in the GPU implementations; for which reason we do not consider them when calculating the speedups (it is sufficient to keep in mind that the overall time necessary for the memory management on the GPU is about 2 times slower than on the CPU). It is important to note that these times for the GPU codes include also the data movement from/to the device; therefore we defined them as* TransferTime* in Tables 3 and 4.

The* TimePerStep* of course increases with the size of the system, in both the sequential and parallel implementations. However, it becomes smaller when launching more simulation instances in the parallel implementation because the total execution time increases less than the number of iterations computed. This means that the device is increasingly exploited more efficiently when launching more thread blocks which hide the latencies caused by sequential dependence and memory stores and loads. This is clear considering the cases 64-32, 52-128, and 56-128 of Table 4. GTX-580 is equipped with 16 SMs composed of 32 cores each, K20 is equipped with 13 SMs composed of 192 cores each, and GTX-Titan is equipped with 14 SMs with the same number of cores as K20. We can see that the ratio between GTX-580 and K20 is about 3, while with GTX-Titan is about 4.6. Besides other aspects as the GPU and Memory Clock rates, these two devices allow using of 4 times the core of the GTX-580.

The implementation scales well when augmenting the number of compartments: in Table 3 we can see that, for a fixed number of simulation instances (i.e., the same number of blocks as 64-32), increasing the system dimension does not affect the speedup achievable.

Moreover, due to the discussed trend of the time per iteration, the speedup increases with the number of simulation instances launched.

The K20 and GTX-Titan devices are equipped with 6 GB of main memory with respect to the 1.5 GB provided by GTX-580; therefore they would be able to execute in theory about 100 concurrent simulations (i.e., the number of blocks). However, this number is limited by the fact that using more threads means using more device memory and, more important, the use of more threads has an impact on the cache miss rate. This is the reason why moving from 52 to 78 blocks on the K20 and from 56 to 84 for GTX-Titan does not result in an improved speedup. The number of blocks launched was decided considering multiples of the number of SMs that are 13 for K20 and 14 for GTX-Titan. For this reason the best achievable result is therefore the 57.4 achieved with the GTX-Titan.

Two final considerations can be made. In a cluster equipped with accelerators we can exploit them and also the available cores by launching at a time both the implementations, paying attention to selecting different random numbers among them. Then, the results seem to suggest that, for this algorithm, it is better to spend money to buy less cluster nodes but equipped with accelerators. This is true for systems that fit in the memory of a GPU, that is, 6 GB for K20 and GTX-Titan, and can be at most 12 for the K40 device. Otherwise the usage of accelerator is no more feasible.

## 8. Conclusions

In this paper we presented two parallelization approaches of the STAUCC simulator, a tool for the efficient stochastic simulation of RD processes in crowded environments based on the S*τ*-DPP algorithm. To this end, we considered a model where gene activation is triggered by transcription factors that diffuse in a crowded environment.

The execution of large experiments with STAUCC, as in general for the RD-based stochastic systems, is very time-consuming. For this reason we analysed how the characteristics of the algorithm can be exploited to accelerate the simulation of the system's evolution using two different parallelization approaches, that is, using message passing techniques based on MPI and GPU computing based on the CUDA architecture.

The major limit of the first parallelization approach is that the exploitation of all the cores within a single node of a cluster yields to good performance if the MPI implementation is smart enough to exploit the shared memory to perform the communications or a hybrid MPI-OpenMP implementation is exploited, whereas the exploitation of multiple nodes is suitable only for large systems and using at least an Infiniband interconnection network.

The use of GPU-based accelerators instead provides very high performance figures, but the amount of main memory of present devices (i.e., up to 12 GB) limits the size of biological models that can be simulated using them. In conclusion, a proper exploitation of a heterogeneous HPC cluster has to consider the combined use of the different nodes and a parallel approach (CPU or GPU based depending on the available compute capabilities) within each node.

Two are the future directions. The first one is that we will experiment the use of the Xeon Phi accelerators, to compare the performance with those achieved by the CUDA devices. The second one is related to the stochasticity of the experiments. So far we considered only the objective to minimize the time to solution. But the CUDA implementation of STAUCC allows us to consider the use of less powerful but more energy efficient devices as those based on GPU+ARM, in order to evaluate also the objective to minimize the energy to solution indicator.

## Figures and Tables

**Algorithm 1:**
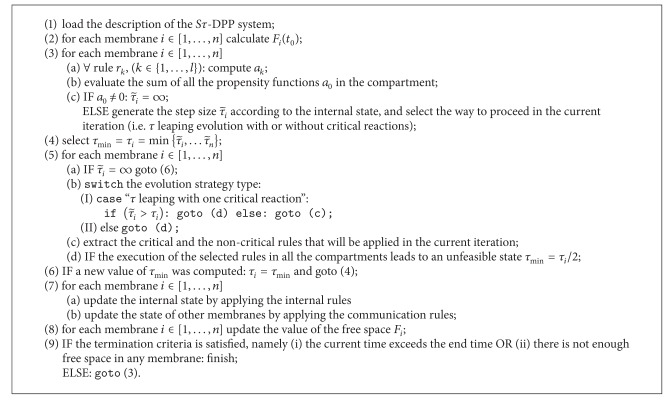
The pseudocode of the *Sτ*-DPP algorithm.

**Table 1:** List of rules defining reaction and diffusion processes. (*x*, *y*) are the compartment's coordinates, *n* ∈ {−1,0, 1}, and *z* ∈ {1,2, 3,4}.

Process	Rule
Activation of G_1_ mediated by F_1_	G_1_ + F_1_ →G_1_^+^
Dissociation of F_1_ from G_1_	G_1_^+^ → G_1_ + F_1_
Activation of G_2_ mediated by F_1_	G_2_ + F_1_ → G_2_^+^
Dissociation of F_1_ from G_1_	G_2_^+^ → G_2_ + F_2_
Inhibition of G_1_ mediated by F_2_	G_1_ + F_2_ → G_1_^−^
Dissociation of F_2_ from G_1_	G_1_^−^ → G_1_ + F_2_
Inhibition of G_2_ mediated by F_2_	G_2_ + F_2_ → G_2_^−^
Dissociation of F_2_ from G_2_	G_2_^−^ → G_2_ + F_2_
Activation of G_1_ mediated by F_3_	G_1_ + F_3_ → G_1_^+^
Dissociation of G_1_ from F_3_	G_1_^+^ → G_1_ + F_3_
Inhibition of G_2_ mediated by F_3_	G_2_ + F_3_ → G_2_^−^
Dissociation of F_3_ from G_2_	G_2_^−^ → G_2_ + F_3_
Inhibition of G_1_ mediated by F_4_	G_1_ + F_4_ → G_1_^−^
Dissociation of G_1_ from F_4_	G_1_^−^ → G_1_ + F_4_
Activation of G_2_ mediated by F_4_	G_2_ + F_4_ →G_2_^+^
Dissociation of F_4_ from G_2_	G_2_^+^ → G_2_ + F_4_
Diffusion from (*x*, *y*) to (*x* + *nx*, *y* + *ny*)	F_*z*_^*x*,*y*^ → F_*z*_^*x*+*nx*,*y*+*ny*^

**Table 2:** Evaluation of the *TimePerStep* execution times: in milliseconds for the sequential version and speedup values using up to 4 nodes of the first cluster with Infiniband connection.

Number of compartments	Number of cores						
	1	2	4	8	16	24	32
256	2.9 ms.	1.86	2.80	3.50	3.20	2.08	2.12
1024	14.6 ms.	1.81	3.90	5.30	6.47	7.03	7.54
4096	63.2 ms.	1.87	3.70	6.10	10.40	15.70	19.80

**Table 3:** Evaluation of the execution times considering the node of the second cluster equipped with the Intel Xeon E5645 CPU and the GTX-580 device. Speedup values are computed considering the *TimePerStep* values.

Number of compartments	Blocks thread	*TransferTime *(ms)	*TimePerStep* (ms)	Speedup
256	Seq.	81.2	2.1	—
	1-32	163.7	6.5	0.3
	32-32	170.8	0.4	5.8
	64-32	159.8	0.2	12.4
	128-32	186.7	0.1	22.6
	256-32	162.7	0.1	24.5

1024	Seq.	82.5	7.6	—
	1-32	193.5	22.1	0.3
	32-32	179.1	1.4	5.5
	64-32	188.6	0.6	12.0
	128-32	182.2	0.4	20.4

4096	Seq.	88.9	29.9	—
	1-32	175.6	80.4	0.4
	32-32	177.0	4.9	6.1
	64-32	205.0	2.4	12.3

**Table 4:** Evaluation of the execution times considering the three different CUDA devices available on the second cluster nodes for the 4096-compartment system. The number of blocks we consider is multiple of the number of SMs available on these devices. Speedup values are computed considering the *TimePerStep* values.

Device	Blocks threads	*TransferTime* (ms)	*TimePerStep* (ms)	Speedup
CPU	—	88.9	29.9	—

GTX-580	32-32	177.0	4.9	6.1
	64-32	205.0	2.4	12.3

K20	13-128	198.6	2.9	10.2
	26-128	182.7	1.5	20.5
	52-128	189.9	0.8	37.8
	78-128	208.6	0.9	32.8

GTX-Titan	14-128	137.4	2.2	13.5
	28-128	136.1	1.1	27.6
	56-128	139.2	0.5	57.4
	84-128	146.5	0.6	48.1

## References

[1] Wang Z, Luo J, Fu G, Wang Z, Wu R (2013). Stochastic modeling of systems mapping in pharmacogenomics. *Advanced Drug Delivery Reviews*.

[2] Cook DL, Gerber AN, Tapscott SJ (1998). Modeling stochastic gene expression: implications for haploinsufficiency. *Proceedings of the National Academy of Sciences of the United States of America*.

[3] Sturrock M, Hellander A, Matzavinos A, Chaplain MAJ (2013). Spatial stochastic modelling of the Hes1 gene regulatory network: intrinsic noise can explain heterogeneity in embryonic stem cell differentiation. *Journal of the Royal Society, Interface/The Royal Society*.

[4] Paulsson J (2005). Models of stochastic gene expression. *Physics of Life Reviews*.

[5] Tan C, Saurabh S, Bruchez MP, Schwartz R, Leduc P (2013). Molecular crowding shapes gene expression in synthetic cellular nanosystems. *Nature Nanotechnology*.

[6] Zimmerman SB, Trach SO (1991). Estimation of macromolecule concentrations and excluded volume effects for the cytoplasm of *Escherichia coli*. *Journal of Molecular Biology*.

[7] Minton AP (2001). The influence of macromolecular crowding and macromolecular confinement on biochemical reactions in physiological media. *The Journal of Biological Chemistry*.

[8] Minton AP (1983). The effect of volume occupancy upon the thermodynamic activity of proteins: some biochemical consequences. *Molecular and Cellular Biochemistry*.

[9] Li G, Berg OG, Elf J (2009). Effects of macromolecular crowding and DNA looping on gene regulation kinetics. *Nature Physics*.

[10] Beg QK, Vazquez A, Ernst J (2007). Intracellular crowding defines the mode and sequence of substrate uptake by *Escherichia coli* and constrains its metabolic activity. *Proceedings of the National Academy of Sciences of the United States of America*.

[11] Richter K, Nessling M, Lichter P (2007). Experimental evidence for the influence of molecular crowding on nuclear architecture. *Journal of Cell Science*.

[12] Mosca E, Cazzaniga P, Pescini D, Mauri G, Milanesi L (2011). Modelling spatial heterogeneity and macromolecular crowding with membrane systems. *Membrane Computing*.

[13] Mosca E, Merelli I, Milanesi L, Clematis A, D’Agostino D A parallel implementation of the S*τ*-DPP stochastic simulator for the modelling of biological systems.

[14] Pasquale G, Maj C, Clematis A A CUDA implementation of the Spatial TAU-leaping in Crowded Compartments (STAUCC) simulator.

[15] Kerr RA, Bartol TM, Kaminsky B (2008). Fast Monte Carlo simulation methods for biological reaction-diffusion systems in solution and on surfaces. *SIAM Journal on Scientific Computing*.

[16] Andrews SS (2012). Spatial and stochastic cellular modeling with the smoldyn simulator. *Methods in Molecular Biology*.

[17] Boulianne L, Al Assaad S, Dumontier M, Gross WJ (2008). GridCell: a stochastic particle-based biological system simulator. *BMC Systems Biology*.

[18] Hepburn I, Chen W, Wils S, de Schutter E (2012). STEPS: efficient simulation of stochastic reaction-diffusion models in realistic morphologies. *BMC Systems Biology*.

[19] Fange D, Mahmutovic A, Elf J (2012). MesoRD 1.0: stochastic reaction-diffusion simulations in the microscopic limit. *Bioinformatics*.

[20] Oliveira RF, Terrin A, di Benedetto G (2010). The role of type 4 phosphodiesterases in generating microdomains of cAMP: large scale stochastic simulations. *PLoS ONE*.

[21] Tolle DP, le Novere DP (2006). Particle-based stochastic simulation in systems biology. *Current Bioinformatics*.

[22] Merelli I, Pescini D, Mosca E (2011). Grid computing for sensitivity analysis of stochastic biological models. *Parallel Computing Technologies (PaCT)*.

[23] Aldinucci M, Coppo M, Damiani F, Drocco M, Torquati M, Troina A On designing multicore-aware simulators for biological systems.

[24] Li H, Petzold L (2010). Efficient parallelization of the stochastic simulation algorithm for chemically reacting systems on the graphics processing unit. *International Journal of High Performance Computing Applications*.

[25] Macchiarulo L A massively parallel implementation of Gillespie algorithm on FPGAs.

[26] Gillespie DT (2007). Stochastic simulation of chemical kinetics. *Annual Review of Physical Chemistry*.

[27] Cao Y, Gillespie DT, Petzold LR (2006). Efficient step size selection for the tau-leaping simulation method. *The Journal of Chemical Physics*.

[28] Chickarmane V, Troein C, Nuber UA, Sauro HM, Peterson C (2006). Transcriptional dynamics of the embryonic stem cell switch. *PLoS Computational Biology*.

[29] Kærn M, Elston TC, Blake WJ, Collins JJ (2005). Stochasticity in gene expression: from theories to phenotypes. *Nature Reviews Genetics*.

[30] van Zon JS, Morelli MJ, Tǎnase-Nicola S, ten Wolde PR (2006). Diffusion of transcription factors can drastically enhance the noise in gene expression. *Biophysical Journal*.

[31] Salwinski L, Eisenberg D (2004). *In silico* simulation of biological network dynamics. *Nature Biotechnology*.

